# The Effects of Question Prompts and Worked Examples on Primary School Students’ Scientific Achievement, Argumentation Skills, Motivation, and Cognitive Load

**DOI:** 10.3390/bs16030335

**Published:** 2026-02-27

**Authors:** Chang Xu, Jinghan Zhu, Yilin Wang, Yafeng Zheng

**Affiliations:** 1 School of International Education, Shandong University, Jinan 250100, China; changxu@sdu.edu.cn; 2 Institute for Advanced Studies in Education, Shandong University, Jinan 250100, China; 3 Faculty of Education, Beijing Normal University Zhuhai Campus, Zhuhai 519000, China; 4 Department of Education, Ocean University of China, Qingdao 266100, China; 5 Center for Educational Science and Technology, Beijing Normal University Zhuhai Campus, Zhuhai 519000, China

**Keywords:** scientific argumentation, question prompts, worked examples, primary school students

## Abstract

It has been widely demonstrated that primary school students face cognitive obstacles in scientific argumentation and often employ ineffective strategies. Scaffolding can effectively guide the scientific argumentation process, enhance its logical rigor, and facilitate students’ reflective thinking during argumentation. The purpose of this study is to examine the effects of question prompts and worked examples on primary school students’ scientific argumentation skills, alongside their scientific achievement, learning motivation and cognitive load. Using a quasiexperimental design, this study involved 68 fourth-grade students and compared the effects of two types of scaffolding: question prompts and worked examples. The results show that both scaffolding strategies exerted positive effects on students’ scientific argumentation skills, scientific achievement and learning motivation. More importantly, the worked examples were significantly more effective than the question prompts in enhancing scientific argumentation skills, particularly in terms of evidence integration and logical reasoning, and they provided greater assistance to students with low levels of prior knowledge. Finally, the worked examples group exhibited significantly lower extraneous cognitive loads than the question prompts group did. This study provides empirical evidence for optimizing the scaffolding design of primary scientific argumentation teaching, confirming that worked examples offer more efficient and adaptive support for novice learners in primary schools in a short time period. From a long-term developmental perspective, it is necessary to gradually fade the support of worked examples and transition to question prompts in scientific argumentation instruction, so as to prompt students to invest more cognitive effort and foster their independent argumentation and critical thinking abilities. These findings have important implications for advancing science curriculum reform and designing targeted instructional interventions.

## 1. Introduction

Scientific argumentation has gradually become a core element of scientific thinking and is formally incorporated into the national science curricula of various nations (MOE of China, 2022; NRC, 2012). However, some studies have reported that the implementation of scientific argumentation activities in primary school still faces a number of challenges. The construction of a quality argument is not a simple cognitive task but rather a complex skill that is particularly difficult for students at this level (Sandoval & Millwood, 2005). One major area of struggle lies in the effective use of evidence, where students often fail to properly evaluate and select valid evidence to support their claims, even when such evidence is presented to them (Maloney, 2007). Students tend to default to constructing their own explanations rather than grounding their claims in data (S. S. Lin & Mintzes, 2010). Furthermore, their critical thinking development is hindered, as these young students are generally unable to identify the inherent constraints or limitations of a given argument (Kyza, 2009), a shortcoming that underscores a notable gap in their metacognitive awareness of the argumentation structure. This gap is manifested in students’ lack of capacity to select and organize evidence, logically link evidence to claims, and critically evaluate the rationality of their own arguments.

In response to the aforementioned challenges, researchers have recommended the construction of appropriate instructional scaffolding to support primary school students’ learning of scientific argumentation (T. C. Lin et al., 2012). Scaffolding refers to the instructional support that is provided to learners to enable them to gradually master learning tasks that they would not be able to complete independently (Fischer et al., 2022; M. C. Kim & Hannafin, 2011). Question prompts and worked examples are two common types of scaffolding for beginner learners (Kern & Crippen, 2017). Question prompts are reflective strategies that use carefully designed questions to guide, deepen, and scaffold students’ thinking (S. J. Lee et al., 2011). Worked examples usually include a clear problem statement and its solution, along with detailed intermediate steps to show the reasoning or process logic connecting the problem (Renkl, 2014). Although both types of scaffolds have been proven to be generally effective in cultivating students’ argumentation skills, existing research reveals an uneven distribution of focus across educational stages. Most studies concentrate on secondary and university students, while the differential effects of these two scaffolding types on scientific argumentation among primary students remain unclear. Therefore, this study is aimed at addressing this gap by employing a quasiexperimental design to directly compare the differential effects of question prompts and worked examples on the scientific achievement, argumentation skills, motivation, and cognitive load of primary school students. To empirically examine these targeted effects and operationalize the core objectives of the study, four specific research questions are formulated as follows.

Q1. What are the differences in the effects of question prompt and worked example scaffolding on primary school students’ scientific achievement?

Q2. What are the differences in the effects of question prompt and worked example scaffolding on primary school students’ scientific argumentation skills?

Q3. What are the differences in the effects of question prompt and worked example scaffolding on primary school students’ learning motivation?

Q4. What are the differences in the effects of question prompt and worked example scaffolding on primary school students’ cognitive load?

## 2. Literature Review

### 2.1. Scientific Argumentation

In science education, argumentation is defined not as a heated exchange but rather as a logical and rational discourse aimed at uncovering the relationships between ideas and evidence, and it involves the development, evaluation, and validation of scientific knowledge (Driver et al., 2000; Duschl et al., 2007). Students are expected to acquire fundamental skills such as argumentation, the analysis of claims and evidence, and the practical application of critical thinking (Cavagnetto, 2010). In fact, the cultivation of scientific argumentation skills not only enables students to “learn to argue” but also to “argue to learn”—that is, to acquire other added-value competencies through the development of scientific argumentation abilities. The integration of argumentation into science education helps students understand the construction of scientific theories, enhances their critical thinking through cognitive engagement, and promotes a more positive learning attitude among students (C. Y. Tsai, 2018; Venville & Dawson, 2010).

Scientific argumentation ability is a developmentally sequential competency, with research demonstrating distinct developmental priorities across age groups (Kuhn, 2010; McNeill & Krajcik, 2011). For elementary school students, who are in the early stage of formal operational thinking development, the core attainable components of scientific argumentation focus on the basic construction of the claim–evidence–reasoning (CER) triad. In contrast, more complex argumentation components derived from Toulmin’s structure—such as rebuttals, backings, and qualifiers—are cognitively demanding and are identified as developmental priorities for secondary and university students, as these learners possess more advanced logical thinking, content knowledge, and critical analysis abilities (M. Kim & Roth, 2019). During the past decade, the teaching of scientific argumentation in schools has been largely informed by McNeill and Krajcik’s (2011) claim–evidence–reasoning (CER) framework, through which students find evidence for a claim and use reason to determine how the evidence connects to the claim. CER is a simplification of the more complex Argument Pattern of Toulmin (Toulmin, 1958), which includes six key components: claim, data, warrant, rebuttal, backing, and qualifier. As an established approach in science curricula, the CER framework’s clear and explicit methodology has been proven effective, as it enables students to rapidly comprehend the logical structure of scientific argumentation and substantially enhances the quality of their arguments (Tyler et al., 2017; W. Lee et al., 2025; Novak & Treagust, 2018; Omarchevska et al., 2022). Consequently, the CER framework is employed in this study to implement scientific argumentation activities for primary students and further explore the integration of effective scaffolding to help students achieve better learning outcomes.

### 2.2. Question Prompts

Question prompts, which are a type of reflective strategy, can be used to guide learners to focus on specific tasks, articulate their thoughts, and reflect on their learning processes (Ge & Land, 2003; Law & Chen, 2016). Question prompts are usually categorized into planning prompts, activity prompts, and monitoring prompts (Davis, 2000), which are suitable for most learning content and learning activities. The use of question prompts has been found to support students’ knowledge integration (Davis, 2000), academic achievement (Cai et al., 2025), and problem-solving (Tawfik et al., 2021). Previous research has found positive effects of question prompts on science learning. For example, in a study on children’s scientific experiment learning, Theobald et al. (2024) designed prompts to guide learners to reflect on potential cognitive conflicts between their initial beliefs and scientific concepts, and found that such prompts significantly enhanced children’s conflict monitoring ability and facilitated their revision of misconceptions about water displacement. Similar positive outcomes have been identified in other relevant studies (Law & Chen, 2016). However, these studies have mostly focused on the role of reflection prompts in scientific knowledge acquisition and problem-solving, with limited attention to their effects on scientific argumentation. Existing research exploring the impact of question prompts on argumentation skills has predominantly targeted college students (Rebello et al., 2013; Tawfik et al., 2021; P. S. Tsai & Tsai, 2013), whereas their effects on primary school students’ argumentation skills and learning motivation remain underexplored. Venville and Dawson (2010) further pointed out that even secondary school students initially demonstrate low-level argumentation (mostly claims only or claims with simple data) without targeted scaffolding, indicating that younger learners may require more structured prompt support.

According to Piaget’s (1950) stages of cognitive development, primary school students (aged 7–11) are in the concrete operational stage, lacking the abstract thinking ability of older learners who are in the formal operational stage and thus requiring more concrete and explicit scaffolding. In addition, primary students have lower domain-specific prior knowledge and lack training in argumentation skills. Previous meta-analyses have also found that the effects of scaffolding vary across different educational levels (Belland et al., 2017). Investigating scaffolding effects in this group can provide empirical evidence for designing age-adaptive scientific argumentation instruction, with specific practical implications for primary science education.

### 2.3. Worked Examples

Worked examples, rooted in example-based learning theory and originating from mathematics, present learners with expert problem–solution pairings (Crippen & Earl, 2007; Hoogerheide et al., 2014). In well-structured domains, such solutions build learners’ understanding of problem-solving and provide predefined scripts and standardized procedures for solution implementation. According to cognitive load theory, worked examples help reduce learners’ extraneous cognitive load, which refers to the unnecessary mental burden imposed by irrelevant instructional or task-related factors rather than the inherent complexity of the learning content itself (Sweller, 2011). This reduction frees up working memory resources and enables students to construct problem-solving schemas (Renkl, 2014; Sweller & Cooper, 1985). Research on worked examples has consistently shown that it is more effective for novice learners (Van Gog & Rummel, 2010; Kollar et al., 2014). In the field of science education, previous studies have investigated the role of worked examples in supporting the science process skills of primary students (Solé-Llussà et al., 2021; Solé-Llussà et al., 2022) and the argumentative essay writing of college students (Latifi et al., 2023). However, their impact on the learning of scientific argumentation among primary students remains unclear. Key differences in age, cognitive development, and subject domains across participant groups are likely to lead to variations in scaffolding effectiveness (Kern & Crippen, 2017). As such, the conclusions from these existing studies cannot be directly generalized to primary school students’ engagement in scientific argumentation activities.

### 2.4. The Moderating Role of Prior Knowledge

A large body of prior research has shown that prior knowledge is regarded as a potential moderator of the effectiveness of various scaffolding strategies. However, whether high- or low-knowledge learners benefit most from instructional support seems to be unclear. For example, Kollar et al. (2014) found that worked examples were particularly effective for students’ acquisition of mathematical argumentation skills with higher prior knowledge. One explanation could be that students with high prior knowledge are more likely to distinguish relevant from irrelevant information and are better able to integrate new information in existing schemata. However, research on the “expertise reversal effect” has yielded contradictory findings (Kalyuga et al., 2012). This theory posits that supporting expert learners with information they already have in long-term memory may be redundant and cause additional extraneous cognitive load (Sweller, 2011). Consistent with this theory, Großmann and Wilde (2019) found that worked examples were more effective for students with low prior knowledge in complex problem-solving processes.

Similar inconsistencies have been reported regarding the moderating role of prior knowledge in the effectiveness of question prompts. Specifically, studies have shown that students with a higher level of prior knowledge demonstrated better learning performances in secondary school physics learning (Moser et al., 2017). In contrast, a meta-analysis by Belland et al. (2017) reported that scaffolding interventions exerted equivalent effects on high- and low-achieving students. Collectively, these conflicting findings highlight that it is crucial to account for learners’ domain-specific prior knowledge of the target topic when evaluating the effectiveness of instructional scaffolds.

## 3. Methods

### 3.1. Participants

In this study, 100 fourth-grade students (from two regular classes with 50 students each) from a public primary school in Guangdong Province, China, were selected as research participants; this class size is typical of primary education in mainland China. All the students were between the ages of 10 and 11 years and were recruited from two naturally formed parallel classes at the school. One class was designated the question prompts group, and the other was designated the worked examples group. Prior to the experiment, a preliminary survey confirmed that none of the participating students had received any form of specialized training in scientific argumentation. Both groups had been instructed by the same experienced science teacher. Thirty-two students were excluded from data analysis for incomplete experimental participation or excessive questionnaire missing values (≥20% on any scale). Ultimately, 68 valid datasets were obtained for analysis: 34 from the question prompts group (16 females) and 34 from the worked examples group (17 females). The basic characteristics, such as sample size and sex ratio, of the two groups were comparable, thus meeting the fundamental requirements of experimental design.

### 3.2. Procedure

**Figure 1:**
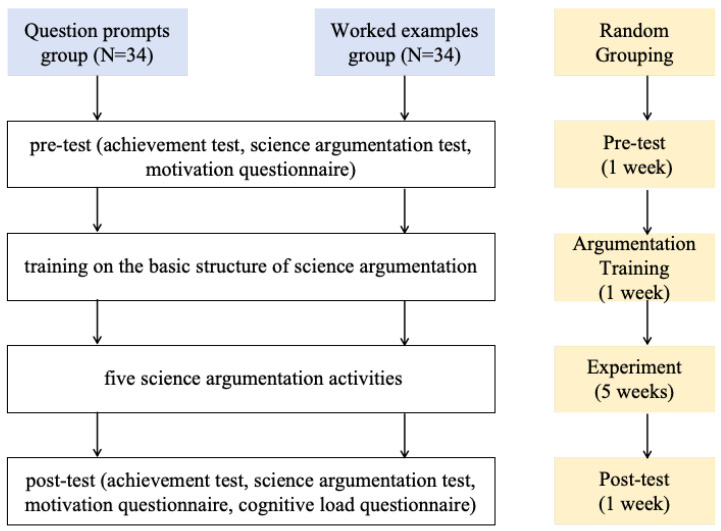
Experimental procedure.

This study utilizes a quasiexperimental design, as illustrated in Figure 1. In Week 1, the science teacher collected data on students’ scientific knowledge, argumentation skills, and learning motivation via paper-and-pencil tests and questionnaires. In Week 2, the teacher explained the basic constituent elements of argumentation and their organizational structure to the students. The teacher explained that a claim is a declarative statement answering a research question (e.g., “Sandstone contains quartz and feldspar”), evidence consists of observable data supporting a claim (e.g., color and luster of grains), and reasoning is the logical explanation connecting evidence to a claim (e.g., “Different colored grains indicate different minerals”). A completed worked example of sandstone analysis was provided to demonstrate how these three components form a coherent argument. From Week 3 to Week 7, the teacher organized students from the two classes to learn the content of a unit entitled Rocks and Soil, which is the third unit of the fourth-grade lower-semester primary school science curriculum. The unit comprised a total of five lessons, namely “Exploring the Characteristics of Different Types of Rocks”, “Exploring the Composition of Rocks”, “Exploring the Composition of Soil”, “Comparing Different Soils”, and “The Relationship Between Rocks, Sand, and Clay”. In Week 8, the teacher collected data on students’ scientific knowledge, argumentation skills, and learning motivation using the same tests and questionnaires as those in the pretest. Additionally, students’ cognitive load was collected via a separate questionnaire.

The students from each of the two classes were assigned different scaffolding strategies. The question prompts, which were standardized and identical for all students in this group, were presented on learning worksheets in the form of logically progressive question chains. This format is aligned with the learning habits of fourth-grade primary school students, who are already accustomed to the integration of written worksheet guidance and teachers’ oral instructional support in their regular science classrooms. Both scaffolding strategies were implemented following five phases: New Lesson Introduction, Knowledge Exploration, Claims Formulation, Evidence Collection, and Reasoning and Argumentation. As an example (shown in Table 1), in the lesson “Exploring the Composition of Rocks”, during the New Lesson Introduction phase, the teacher established the same rock inquiry context for both groups, raised core questions, clarified the inquiry task, and refrained from directly providing research methods or demonstrations. In the Knowledge Exploration phase, the teacher provided introductory materials on rock types to the question prompts group and guided their reflection via metacognitive prompts, while presenting rock classification diagrams, characteristic comparison tables, and expert tips to the worked examples group, and organizing targeted discussions for both groups. For the Claims Formulation phase, the teacher created cognitive conflicts for the question prompts group by presenting opposing viewpoints, provided sandstone research claim examples to the worked examples group, and guided students in both groups to state classification claims in a standardized manner. During the Evidence Collection phase, the teacher designed step-by-step question chains for the question prompts group, offered scientists’ inquiry procedures and data to the worked examples group, and instructed students in both groups to complete the rock observation record form. In the final Reasoning and Argumentation phase, the teacher guided the question prompts group in evidence-based reasoning through multi-dimensional question chains, demonstrated the claim–evidence–reasoning tripartite framework to the worked examples group, and assisted students in both groups in drawing scientific conclusions.

### 3.3. Measuring Tools

#### 3.3.1. Achievement Test

The achievement test was developed to assess students’ understanding of the composition and classification of rocks and soil, as well as their evolutionary relationships. The test was developed based on the China National Curriculum 2022 and the school’s 4th grade science subject syllabus. It consisted of 10 single-choice items, 1 multiple-choice item, and 1 reverse-coded item and focused on students’ conceptual understanding of two related topics: 6 items on rocks and 6 items on sand and soil. Sample items include “Which mineral is the softest among quartz, feldspar, and mica?” and “Which type of soil is suitable for growing lotus and reed?”. Students are awarded 8 points for each correctly answered single-choice item and 10 points for each correctly answered reverse-coded item or multiple-choice item; thus, the maximum score is 100 points. The development process for this assessment instrument strictly adhered to the following procedures: First, researchers specializing in science education drafted the initial version. Senior primary school science teachers with years of teaching experience were subsequently invited to conduct professional reviews. The final version was confirmed after three rounds of revisions. To ensure the scientific rigor and reliability of the instrument, the Cronbach’s alpha coefficient was used to rigorously measure the internal consistency of the items, yielding a coefficient value of 0.843.

#### 3.3.2. Scientific Argumentation Assessment

The scientific argumentation assessment (SAA) is an open-ended scenario-based instrument that was developed to measure the level of students’ argumentation skills. The SAA contains two scenario-based argumentation questions pertaining to rocks and soil. The students were required to generate their own claim, evidence, and reasoning for each scenario-based question. The assessment rubric uses the scientific argumentation evaluation framework proposed by McNeill and Krajcik (2011), in which students’ scientific argumentation competence is decomposed into four core components: claim construction, evidence, reasoning, and rebuttal. Given their age characteristics, rebuttal competence is relatively challenging for primary school students. Consequently, this study ultimately selects three dimensions—claim, evidence, and reasoning—to assess students’ argumentation performance, and the specific criteria used are presented in Table 2. The assessment was constructed by a primary school science teacher and a postdoctoral fellow, and the interrater reliability score was 0.91.

#### 3.3.3. Learning Motivation Questionnaire

We employed a five-point Likert scale questionnaire adapted from Christopoulos and Mystakidis (2023) to assess the impact of scaffolding on changes in students’ learning motivation. The questionnaire was further divided into two dimensions: “relevance (4 items)” and “satisfaction (3 items)”. Specifically, “relevance” reflects the extent to which learners perceive connections between the course content and their prior knowledge, current needs, as well as future applications, whereas “satisfaction” denotes the sense of enjoyment and accomplishment that learners experience during and after the course. Examples of the items used include “I can link the course content to daily observations, experiences, or reflections” and “I am highly interested in the course content”. The questionnaire used a 5-point Likert scale, which showed acceptable reliability: the Cronbach’s alpha was 0.792 for the total scale and 0.777 and 0.703 for the “relevance” and “satisfaction” dimensions, respectively. Exploratory factor analysis (EFA) via Principal Axis Factoring with Oblimin rotation showed KMO = 0.77, Bartlett’s test significance (χ^2^ = 110.20, df = 21, *p* < 0.001); parallel analysis and a scree plot supported a two-factor solution (68.74% total variance explained). Factor loadings ranged 0.43–0.79, with no cross-loadings > 0.30, confirming the two-dimensional structure.

#### 3.3.4. Cognitive Load Questionnaire

We adopted a 5-point Likert scale adapted from Quintero-Manes and Vieira (2025) to measure the changes in students’ cognitive load. The instrument comprises two dimensions: “intrinsic cognitive load (4 items)” and “extraneous cognitive load (3 items)”. Specifically, “intrinsic cognitive load” reflects the difficulty that students encounter when independently engaging with course content, whereas “extraneous cognitive load” denotes the exertion required of them to complete learning tasks and achieve corresponding objectives. Sample items include “answering questions in previous learning activities was quite challenging” and “I need to exert considerable effort to complete learning tasks or achieve objectives”. The questionnaire demonstrated acceptable reliability (Cronbach’s α = 0.759 for the total scale; 0.781 and 0.742 for the “intrinsic cognitive load” and “extraneous cognitive load” dimensions, respectively). Exploratory factor analysis (EFA) via Principal Axis Factoring with Oblimin rotation showed KMO = 0.76, Bartlett’s test significance (χ^2^ = 169.23, df = 21, *p* < 0.001); parallel analysis and a scree plot supported a two-factor solution (73.53% total variance explained). Factor loadings ranged 0.58–0.97, with no cross-loadings > 0.30, confirming the two-dimensional structure.

### 3.4. Data Analysis

To evaluate the differential effects of question prompt (QP) and worked example (WE) scaffolding, we conducted a series of 2 (Time: Pretest vs. Posttest) × 2 (Group: QP vs. WE) mixed-measures ANOVAs on four dependent variables: scientific achievement, scientific argumentation ability, learning motivation, and cognitive load. Prior to formal statistical analysis, normality of distribution was assessed for all variables, and parametric mixed-measures ANOVAs were retained for those that met the normality assumption. For several variables where the homogeneity of variance assumption was violated, Welch’s correction was applied to adjust the test statistics accordingly. For variables that yielded a significant Time × Group interaction effect, follow-up simple effects analyses were conducted: paired samples *t*-tests were used to examine within-group changes from pretest to posttest, and independent samples *t*-tests (with Welch’s correction applied where appropriate) were performed to compare between-group differences at each time point.

## 4. Results

### 4.1. Comparison of the Achievement Tests Between the Two Groups

As shown in Table 3, for achievement test scores, the ANOVA revealed a significant main effect of time, indicating that the students in both conditions significantly improved their domain knowledge during the period between the pretest and the posttest. The results of the paired samples *t*-test indicated that the posttest scores of both QP (*M*_pre_ = 48.76, *M*_post_ = 72.12; *t*(33) = 7.62, *p* < 0.001) and WE (*M*_pre_ = 45.41, *M*_post_ = 71.71; *t*(33) = 7.68, *p* < 0.001) were significantly higher than their respective pretest scores, with a large effect size observed for both variables. Specifically, the Cohen’s *d* value was 1.31 for QP and 1.32 for WE, shown in Table 4, which suggests comparable growth in scientific knowledge across conditions. The main effect of Group (*p* = 0.468) and the Time × Group interaction (*p* = 0.524) were nonsignificant.

### 4.2. Comparison of the Scientific Argumentation Ability Between the Two Groups

As shown in Table 2, the assessment of scientific argumentation ability encompassed three core dimensions: claim, evidence, and reasoning.

For claim skills, the ANOVA results indicated a significant main effect of Time (*F*(1, 66) = 54.69, *p* < 0.001, *η*^2^*_p_* = 0.45), reflecting that students in both groups achieved significant improvements in claim skills from pretest to posttest. Neither the main effect of Group (*p* = 0.901) nor the Time × Group interaction (*p* = 0.796) reached statistical significance. The results of paired samples *t*-tests revealed significant pretest–posttest gains for QP (*M*_pre_ = 0.21, *M*_post_ = 1.02; *t*(33) = 4.83, *p* < 0.001) and WE (*M*_pre_ = 0.19, *M*_post_ = 1.06; *t*(33) = 5.68, *p* < 0.001), with a large effect size observed for QP (Cohen’s *d* = 0.83) and an even larger effect size for WE (Cohen’s *d* = 0.98).

With regard to evidence skills, significant main effects of Time (*p* < 0.001) and Time × Group interaction (*p* = 0.001) were found. The results of paired samples *t*-tests revealed significant pretest–posttest gains for QP (*M*_pre_ = 0.69, *M*_post_ = 1.34; *t*(33) = 5.95, *p* < 0.001) and WE (*M*_pre_ = 0.46, *M*_post_ = 1.74; *t*(33) = 8.16, *p* < 0.001), with a large effect size observed for QP (Cohen’s *d* = 1.02) and an even larger effect size for WE (Cohen’s *d* = 1.40). Between-group comparisons showed no significant difference at pretest (*t*(66) = 1.99, *p* = 0.051, Cohen’s *d* = 0.48), while the WE group obtained significantly higher posttest scores than the QP group (*t*(66) = −2.54, *p* = 0.014, Cohen’s *d* = 0.62).

For reasoning skills, significant main effects of Time (*p* < 0.001) and Time × Group interaction (*p* = 0.002) were detected. Within-group analyses confirmed significant pretest-to-posttest improvements in the QP group (*M*_pre_ = 0.35, *M*_post_ = 1.02; *t*(33) = 6.06, *p* < 0.001) and the WE group (*M*_pre_ = 0.16, *M*_post_ = 0.73; *t*(33) = 9.25, *p* < 0.001),with a large effect size observed for QP (Cohen’s *d* = 1.04) and an even larger effect size for WE (Cohen’s *d* = 1.59). Between-group *t*-tests revealed significant group differences at both pretest (*t*(59.3) = 2.01, *p* = 0.049, Cohen’s *d* = 0.49) and posttest (*t*(66) = −2.05, *p* = 0.045, Cohen’s *d* = 0.50).

To further explore the impact of the two types of argumentation scaffolds on students with different levels of prior knowledge, the top 27% of the students were classified as the high-prior-knowledge-level group and the bottom 27% as the low-prior-knowledge-level group on the basis of their pretest scientific achievement scores, following an established methodological convention in educational research to balance effect size between groups and sample size adequacy (Wiersma & Jurs, 1985). The Mann–Whitney U test was subsequently used to analyze the differences in scientific argumentation ability improvement between the students in the high- and low-level groups. The results are shown in Table 5. In the QP group, there were no significant differences in the claim (U = 32.000, z = −0.760, *p* = 0.447), evidence (U = 30.500, z = −0.919, *p* = 0.358), and reasoning (U = 37.000, z = −0.316, *p* = 0.752) between the high- and low-prior-knowledge-level students. In the WE group, there were significant differences observed in the evidence (U = 13.500, z = −2.470, *p* = 0.014) between the high- and low-level students, with the low-prior-knowledge-level group demonstrating a greater improvement. No significant differences were found between the two groups in terms of the claim (U = 28.500, z = −1.120, *p* = 0.263) and reasoning (U = 25.500, z = −1.351, *p* = 0.177).

### 4.3. Comparison of the Learning Motivation Between the Two Groups

As shown in Table 3, for relevance, the ANOVA revealed a significant main effect of time (*F*(1, 66) = 28.4, *p* < 0.001, *η*^2^*_p_* = 0.3), with both groups showing significant pretest–posttest improvements. The main effects of Group (*p* = 0.413) and Time × Group interaction (*p* = 0.647) were nonsignificant. The results of paired samples *t*-tests revealed significant pretest–posttest gains for QP (*M*_pre_ = 3.46, *M*_post_ = 4.02; *t*(33) = 4.13, *p* < 0.001) and WE (*M*_pre_ = 3.39, *M*_post_ = 3.86; *t*(33) = 8.16, *p* = 0.001), with a medium-to-large effect size observed for QP (Cohen’s *d* = 0.71) and a medium effect size for WE (Cohen’s *d* = 0.59).

Regarding satisfaction, the ANOVA indicated a significant main effect of time (*F*(1, 66) = 14.32, *p* < 0.001, *η*^2^*_p_* = 0.18), with significant pretest–posttest improvements in both groups. Importantly, a significant Time × Group interaction was observed (*F*(1, 66) = 4.46, *p* = 0.0038, *η*^2^*_p_* = 0.06). The results of paired samples *t*-tests revealed significant pretest–posttest gains for QP (*M*_pre_ = 3.67, *M*_post_ = 3.85; *t*(33) = 1.08, *p* = 0.288) and WE (*M*_pre_ = 3.50, *M*_post_ = 4.16; *t*(33) = 4.66, *p* < 0.001), with a small effect size observed for QP (Cohen’s *d* = 0.19) and a large effect size for WE (Cohen’s *d* = 0.80). Between-group *t*-tests showed no significant differences at pretest (*t*(66) = 1.41, *p* = 0.16) or posttest (*t*(60.8) = −1.48, *p* = 0.14).

### 4.4. Comparison of Cognitive Load Between the Two Groups

To explore the impact of the two types of scaffolds on primary school students’ cognitive load levels, an independent samples t test was used to examine the differences in the cognitive load levels of students from the two classes after the experiment. The results indicate that there was a statistically significant difference in the extraneous cognitive load (*t*(33) = 2.07, *p* = 0.042, Cohen’s *d* = 0.62) between the two groups, with the worked examples group being significantly lower than the question prompts group, while no significant difference was found in intrinsic cognitive load (*t*(33) = 1.62, *p* = 0.109, Cohen’s *d* = 0.78) between the two groups.

## 5. Discussion

In this study, we investigated the different effects of question prompts (QP) and worked examples (WE) on primary school students’ scientific achievement, argumentation skills, motivation, and cognitive load in a classroom environment. Furthermore, we examined the question of whether the effectiveness of the two scaffolding approaches is dependent on students’ prior achievements. The discussion is organized according to these aspects.

The results indicated that both the QP and WE scaffolds significantly improved students’ academic achievement, with no significant difference in the magnitude of improvement between the two groups. This finding confirms that both types of argumentation scaffolds are effective in facilitating students’ understanding and mastery of scientific concepts, which aligns with the core tenets of scaffolded instruction proposed by Vygotsky’s (1978) zone of proximal development theory. The finding is consistent with previous studies (Kern & Crippen, 2017; Venville & Dawson, 2010). Essentially, both QP and WE scaffolds serve as cognitive supports that reduce students’ extraneous cognitive load during scientific learning, thereby enabling them to focus on processing and integrating core scientific concepts For QP scaffolds, the targeted questions guide students to actively reflect on scientific concepts, initiate self-explanation, and construct knowledge. This active engagement promotes deep understanding rather than the superficial memorization of concepts. Similarly, WE scaffolds provide students with well-structured, complete examples that model the correct way to link scientific concepts with phenomena and questions, thereby enhancing their conceptual understanding and application ability.

The positive effects of question prompts (e.g., Tawfik et al., 2021; P. S. Tsai & Tsai, 2013) and worked examples (e.g., Hefter et al., 2019; Vogel et al., 2016) on argumentation skills are well documented in the literature. In line with this documentation, we find that both QP and WE groups improved in scientific argumentation ability (claim, evidence, and reasoning). Notably, the WE scaffold exhibited greater advantages in promoting evidence and reasoning skills, with a significant Time × Group interaction effect—WE students achieved more remarkable improvements in these two dimensions than QP students, and the effect size was larger. Previous research has shown that novices tend to focus on the surface features of problems and struggle to construct the rule-based inferences required to depict the causal relationships underlying the phenomenon (Hmelo-Silver et al., 2007), and this cognitive limitation is amplified in primary school students due to their still-developing sustained attention and logical reasoning abilities, which are key developmental factors in this age group. Worked examples, by providing a clear model of how evidence is selected and connected to a claim through reasoning, offer a concrete schema that students can emulate, making tangible the abstract process of argumentation. In contrast, question prompt scaffolds rely on students’ active reflection and independent construction, which place higher demands on their developing reading skills and ability to maintain attention for independent thinking, and thus may require more cognitive effort and lead to relatively smaller improvements in complex dimensions. The fact that both groups performed equally well in the construction of claims indicates that stating a basic position is a relatively accessible skill. The challenge for primary students, as highlighted in the literature (Maloney, 2007; S. S. Lin & Mintzes, 2010), lies in the subsequent steps, namely, evidence and reasoning, which is precisely where worked examples are the most beneficial.

A further important finding concerns the role of students’ prior knowledge. In the QP group, there was no significant difference in argumentation ability improvement between high- and low-prior-knowledge students, while in the WE group, low-prior-knowledge students showed significantly greater improvements in evidence skills. This suggests that the WE scaffold is more adaptable to low-prior-knowledge students, which contrasts with the findings of previous studies by Kollar et al. (2014). This robustly supports the “expertise reversal effect” (Kalyuga et al., 2001), in which instructional support that is effective for novices becomes redundant for more knowledgeable learners. For high-level learners, scaffolding may impose an unnecessary cognitive load, as such learners already possess existing schemas for problem-solving and require working memory to process redundant information.

This study found that both QP and WE scaffolds significantly enhanced primary school students’ scientific learning motivation in the relevance and satisfaction dimensions. For the relevance dimension, the non-significant main effect of Group and Time × Group interaction indicated that the two scaffolds had comparable promotion effects. This is because both were designed based on scientific content closely related to daily life: QP guided students to actively connect scientific knowledge with daily experiences through targeted questions, while WE naturally reflected the practical application of knowledge through structured examples. A significant Time × Group interaction was observed in the satisfaction dimension: the WE group’s interest in the course content improved significantly with a large effect size, while the QP group showed no statistically significant change. This divergence aligns with the cognitive load results: the WE scaffold reduced the cognitive effort required for argumentation tasks, alleviated learning frustration, and enhanced students’ sense of learning competence, thereby boosting their interest and satisfaction. In contrast, the QP scaffold demanded more cognitive effort for independent argumentation construction; the challenges in this process did not erode students’ original interest, but failed to improve their satisfaction in the short term.

The cognitive load results revealed no significant difference in intrinsic cognitive load between the QP group and the WE group, while the WE group exhibited significantly lower extraneous cognitive load than the QP group. From the perspective of CLT, the primary goal of instructional design is to reduce extraneous cognitive load to optimize cognitive resource allocation for meaningful knowledge construction. The non-significant difference in intrinsic cognitive load indicates that both scaffolds equally and effectively regulated the inherent cognitive burden imposed by scientific argumentation tasks on novice primary school students.

The significant difference in extraneous cognitive load reflects the distinct ways in which the two scaffolds shape students’ cognitive resource allocation, and this difference is the core of their instructional characteristics. The WE scaffold acts as an explicit “cognitive template”, directly modeling the logical connections between claims, evidence and reasoning, and reducing the need for students to independently generate, organize and verify argumentation structures. This design results in lower extraneous cognitive load because students do not need to allocate a large amount of cognitive resources to construct argumentation frameworks from scratch. In contrast, the QP scaffold requires students to allocate more cognitive processing resources to analyze task requirements, connect prior knowledge with new content, and organize logical reasoning, hence the higher extraneous cognitive load in the QP group. In the short term, both scaffolds can effectively promote students’ scientific argumentation learning. However, the lower extraneous cognitive load in the WE group, while beneficial for immediate task completion, may limit the development of students’ independent argumentation ability and knowledge transfer ability in the long run, as it reduces the deliberate cognitive effort required for deep learning and skill construction. From a practical teaching perspective, this result provides refined guidance for primary school science teachers to select and optimize argumentation scaffolds, with the core principle of balancing extraneous cognitive load reduction and the cultivation of independent cognitive ability. Corresponding strategies can be adopted, such as gradually fading in worked examples and adding reflective questions on example logic, to appropriately increase students’ cognitive effort investment on the basis of initially reducing their extraneous cognitive load.

## 6. Conclusions, Limitations and Future Study

In conclusion, this study finds that both question prompts and worked examples effectively improve primary school students’ scientific knowledge mastery with comparable effects. Worked examples, however, show distinct superiority in fostering scientific argumentation skills, significantly enhancing students’ evidence use and reasoning abilities—with this advantage being most pronounced among low-prior-knowledge students. Additionally, worked examples reduce students’ subjective extraneous cognitive load more effectively and boosts their learning satisfaction significantly, a positive affective outcome not seen in the question prompts group. These results confirm that worked examples are a more efficient and supportive scaffolding approach for initial scientific argumentation instruction in primary school, especially suited to young learners’ developmental characteristics and the needs of low-prior-knowledge students. While question prompts elicit more active cognitive engagement critical for long-term independent argumentation and critical thinking development, the hybrid integration of worked examples and reflective question prompts represents a promising direction for future teaching, which can be adaptively tailored to diverse learner profiles and prior knowledge levels to balance cognitive burden reduction and deep learning.

This study has several limitations. First, the reliance on a single classroom within one school limits the generalizability of the findings. Future studies should recruit participants from a broader range of schools and backgrounds to test whether the effects of the two scaffolding types hold across diverse educational and cultural contexts. Second, this study is focused on a direct comparison of two scaffold types; future studies should apply a faded scaffolding approach, where worked examples are gradually replaced by question prompts as students gain proficiency. Finally, combining the strengths of both scaffolds—by embedding reflective question prompts within worked examples for instance—presents a promising pathway for designing even more robust and adaptive learning supports for scientific argumentation.

## Figures and Tables

**Table 1:** The implementation process details of the two scaffolds.

Inquiry Activity Phase	Question Prompts	Worked Examples
New Lesson Introduction	Present rock images to establish inquiry context; pose core questions to guide reflection on rock characteristics without providing specific methods or examples. Example: “When observing a rock, we see different grains or crystals (e.g., quartz, feldspar, mica) with unique properties. Some rocks contain one grain type, others multiple. How can we distinguish them?”	
Knowledge Exploration	Provide introductory materials on rock types; use prompts to guide reflection on limitations of single-characteristic judgment and recognize the need for multi-characteristic observation. Example: “Some minerals look alike—pyrite and gold are both golden. How would you distinguish them? Which represents the true mineral color: surface color or streak?”	Provide cyclic flow diagram of rock classification, characteristic comparison table with expert tips, and counterexamples (e.g., non-lustrous rocks are not necessarily sedimentary). Example: <Expert Tip> When observing pyrite and gold, scientists found that these two minerals have very similar surface colors, both appearing yellow; however, when scientists rubbed pyrite and gold separately on a white unglazed porcelain plate, they discovered that pyrite’s streak color is black, while gold’s streak remains yellow. Therefore, scientists concluded that streak color represents the true color of a mineral.
Claims Formulation	Create cognitive conflicts via opposing viewpoints; require students to state classification claims in complete sentences. Example: “Some believe granite contains quartz and feldspar. Do you agree? State your claim: I ____ (agree/disagree); I believe granite contains ____.”	Provide complete worked example of sandstone research demonstrating how scientists formulate claims; students imitate to propose their own claims. Example: Research question: What minerals does sandstone contain? Proposed claim: Sandstone includes quartz and feldspar two minerals. “1. Research question: What minerals do you think granite contains? My claim/hypothesis: ____” Students directly imitate the structure of the sandstone example to propose their own claims about granite.
Evidence Collection	Design step-by-step question chains to guide systematic observation; require recording results in observation forms; emphasize multi-dimensional evidence collection. Example: “How many differently-colored grains appear on the granite surface? What are their color, transparency, and luster?”	Present scientists’ complete inquiry steps and detailed observation data; students observe materials and complete identical forms by referring to scientists’ procedures. Example: First, use a magnifier to observe how many differently-colored grains appear on the granite surface; Then, observe the color, transparency, and luster of these grains respectively, and record the results in the observation table.
Reasoning and Argumentation	Guide comparative reasoning based on evidence via multi-dimensional question chains; prompt students to write claims, list characteristic evidence and reasoning processes, and draw scientific conclusions. Example: “My revised claim: ____. Evidence: ____. Reasoning: Because granite has ____ colored grains … matching ____ minerals’ characteristics …”	Provide flowchart-style worked example of scientists’ reasoning (claim–evidence–reasoning tripartite structure); demonstrate matching data with characteristics for classification conclusions. Example: My claim: Sandstone contains two minerals, quartz and feldspar. My evidence: According to the summary table, quartz features white surface, white streak, transparent, and glass luster; feldspar features flesh-red surface, white streak, opaque, and glass luster; mica features black surface, colorless streak, translucent, and silky luster. Using a magnifier to observe, sandstone surface has two differently-colored grains: Grain 1 appears white, transparent, and glass luster; Grain 2 appears flesh-red, opaque, and glass luster. My reasoning: 1. Because sandstone surface has two differently-colored grains, according to scientific definition, different colored grains represent different minerals, so sandstone contains two minerals; 2. Through observation, grains on sandstone surface match the characteristics of quartz and feldspar most closely, so sandstone contains two minerals, quartz and feldspar.

**Table 2:** Assessment criteria for scientific argumentation.

Skill	Level 1	Level 2	Level 3	Level 4
Claim	There is no hypothesis related to the proposition or an unclear claim is presented.	The opposing position is generalized, but it lacks specificity or provides unclear referents.	A general claim related to the proposition is presented, but it is not complete.	A clear and concrete generalization related to the proposition is stated.
Evidence	No supporting data is provided or the provided data is irrelevant to the claim.	The data or evidence provided is weak, inaccurate, or incomplete.	The provided data is relevant, but it is not complete.	The supporting data is persuasive, accurate, and relevant to the claim.
Reasoning	No rules or principles are provided.	Fails to connect the data with the claim, or most of the rules and principles are invalid or irrelevant.	The data is explained in some way, but this explanation is not specifically connected to the claim.	The explanation of the data clearly shows how these data support this claim.

**Table 3:** Summary of mixed ANOVA of two groups.

Variable	Source	df	F	*p*	η^2^_p_
Achievement	Time	1	116.76	<0.001 ***	0.64
	Group	1	0.53	0.468	0.01
	Time × group	1	0.41	0.524	0.01
Argumentation Skill					
Claim	Time	1	54.69	<0.001 ***	0.45
	Group	1	0.02	0.901	0.00
	Time × group	1	0.07	0.796	0.00
Evidence	Time	1	101.91	<0.001 ***	0.61
	Group	1	0.65	0.425	0.01
	Time × group	1	10.98	0.001 **	0.14
Reasoning	Time	1	120.72	<0.001 ***	0.65
	Group	1	0.54	0.466	0.01
	Time × group	1	10.25	0.002 **	0.13
Learning Motivation					
Relevance	Time	1	28.84	<0.001 ***	0.3
	Group	1	0.68	0.413	0.01
	Time × group	1	0.21	0.647	0.00
Satisfaction	Time	1	14.32	<0.001 ***	0.18
	Group	1	0.30	0.584	0.00
	Time × group	1	4.46	0.038 *	0.06

* *p* < 0.05; ** *p* < 0.01; *** *p* < 0.001.

**Table 4:** Descriptive statistics results for the two groups.

Variable	Group	Pre		Post	
		M	SD	M	SD
Achievement	QP	48.76	15.31	72.12	13.43
	WE	45.41	14.02	71.71	14.17
Argumentation Skill					
Claim	QP	0.21	0.30	1.02	0.94
	WE	0.19	0.46	1.06	0.80
Evidence	QP	0.69	0.51	1.34	0.57
	WE	0.46	0.47	1.74	0.71
Reasoning	QP	0.35	0.45	1.02	0.69
	WE	0.16	0.32	1.37	0.73
Learning Motivation					
Relevance	QP	3.46	0.67	4.02	0.81
	WE	3.39	0.63	3.86	0.72
Satisfaction	QP	3.67	0.48	3.85	0.96
	WE	3.50	0.49	4.16	0.71
Cognitive Load					
Intrinsic Cognitive Load	QP	-	-	2.13	0.74
	WE	-	-	1.82	0.83
Extraneous Cognitive Load	QP	-	-	2.26	0.54
	WE	-	-	1.95	0.70

QP = question prompts; WE = worked examples.

**Table 5:** Mann–Whitney U test of the high and low groups.

Group	Skill	Group	N	U	Z	*p*
QP	claim	High	9	32.000	−0.760	0.447
		Low	9			
	evidence	High	9	30.500	−0.919	0.358
		Low	9			
	reasoning	High	9	37.000	−0.316	0.752
		Low	9			
WE	claim	High	9	28.500	−1.120	0.263
		Low	9			
	evidence	High	9	13.500	−2.470	0.014 *
		Low	9			
	reasoning	High	9	25.500	−1.351	0.177
		Low	9			

* *p* < 0.05; QP = question prompts; WE = worked examples.

## Data Availability

The data supporting the findings of this study are available from the corresponding author upon reasonable request.
